# Discharge of Acute Coronary Syndrome Patients on Sub-Optimal Dual Anti-Platelet Therapy: A Single Center Experience

**DOI:** 10.21203/rs.3.rs-3425525/v1

**Published:** 2023-10-19

**Authors:** Jeffrey Booker, alexander Nihart, matthew Campen, Eduardo Medrano-Rodriguez, James Blankenship

**Affiliations:** University of New Mexico School of Medicine; University of New Mexico Health Sciences Center; University of New Mexico Health Sciences Center; University of New Mexico School of Medicine; University of New Mexico

**Keywords:** ticagrelor, clopidogrel, dual anti-platelet therapy, therapeutic inertia

## Abstract

**Purpose:**

To identify and quantify the reasons why acute coronary syndrome (ACS) patients undergoing stenting at University of New Mexico Hospital were prescribed sub-optimal dual antiplatelet therapy (DAPT) at discharge, and to identify practice patterns that could potentially lead to improved DAPT treatment for these patients.

**Methods:**

We reviewed electronic medical records and cardiac catheterization records of 326 patients who underwent PCI at UNMH between January 1, 2021, and June 30, 2022 and identified 229 ACS patients who survived until discharge. Demographic and clinical characteristics relevant to P2Y_12_ selection were obtained from a review of medical records. Pharmacists’ notes that documented their efforts to get appropriate insurance coverage and reasons for discharge on clopidogrel rather than ticagrelor were reviewed. Patients discharged on aspirin and clopidogrel underwent review of medical records and cardiac catheterization lab records to determine if the discharge P2Y_12_ drug was appropriate. Reasons for inappropriately discharge on clopidogrel were categorized as cost/insurance, patient preference, concern for daily adherence to a twice-daily medication, and on clopidogrel before PCI and not switched to ticagrelor afterward.

**Results:**

The 229 ACS patients included (38.0%, n = 87) appropriately discharged on ticagrelor/prasugrel, (27.5%, n = 63) appropriately discharged on clopidogrel, (32.8%, n = 75) inappropriately discharged on clopidogrel, and (1.7%, n = 4) not discharged on a P2Y_12_ inhibitor. For patients inappropriately discharged on clopidogrel (n = 75), the most common reasons were cost or lack of insurance (n = 56) and clinical inertia (taking clopidogrel before PCI and maintained on it afterward) (n = 17). Inappropriate DAPT at discharge correlated with lack of insurance (90.5% compared to 39.7% in patients with insurance, P < 0.001) but not with ethnicity.

**Conclusion:**

At the University of New Mexico, a safety-net hospital, increasing financially restricted access to ticagrelor could help up to 24.5% of ACS patients reduce their risk of ischemic events. For patients admitted on clopidogrel DAPT, upgrading to ticagrelor could reduce ischemic risk in 7.4% of ACS patients. Expanding healthcare insurance coverage might redue sub-optimal DAPT coverage.

## Introduction

Since the early 1990’s, dual anti-platelet therapy P2Y_12_ has been the standard of care after coronary stenting to prevent stent thrombosis. Ticlopidine was the first P2Y_12_ drug, but it was replaced in the early 2000’s by clopidogrel. The 2004 American College of Chest Physicians 7th Conference on Anti-thrombotic and Thrombolytic Therapy included a class IA recommendation for the use of clopidogrel over ticlopidine after stenting[1]. In subsequent years, prasugrel and ticagrelor were found in randomized clinical trials to be superior to clopidogrel in preventing ischemic events[2]. However, these trials also demonstrated increased risks of bleeding compared to clopidogrel. The effect of ticagrelor, unlike clopidogrel, on platelet aggregation were also shown to be unaffected by genetic polymorphisms of CYP2C19[3], suggesting less variance of efficacy in different populations. Based on randomized controlled trials and non-randomized cohort studies[4–6], the 2021 ACC/AHA revascularization guidelines[7] and European Society of Cardiology guidelines[8] recommend ticagrelor or prasugrel over clopidogrel for acute coronary syndrome (ACS) patients. Despite this, there has been incomplete adoption of prasugrel/ticagrelor over clopidogrel over the past decade, based on the higher cost of the former, the trade-off between bleeding and anti-thrombotic effects, which are both higher with the former and data from large cohort studies suggest similar efficacy in real-world ACS patients.[9, 10] Furthermore, prasugrel and ticagrelor do not have FDA-approved indications for stable patients, so clopidogrel remains the drug of choice for stable patients undergoing stenting. The choice of optimal P2Y_12_ therapy may be ambiguous for patients with atypical symptoms if it is unclear whether those symptoms represent stable or unstable angina.

Other factors limit use of ticagrelor or prasugrel. These include a history of intracranial hemorrhage, active bleeding, and severe hepatic impairment, conditions that can increase increase risk of fatal bleeding.[11] Furthermore, dyspnea is a known adverse effect of ticagrelor that may lead to substitution of a different P2Y_12_ inhibitor[12].

At the University of New Mexico Hospital (UNMH), recommendations for P2Y_12_ medications after stenting are made by the interventional cardiologists performing the stenting procedure. However, the final decisions regarding discharge medications are typically made by inpatient service cardiologists, often with input from clinical pharmacologists who investigate patients’ insurance coverage and their ability to afford medications. Prasugrel was not on the UNMH formulary during the study period, so consequently when patients could not afford ticagrelor due to lack of insurance coverage or high out-of-pocket co-pay requirements, the cheaper alternative (clopidogrel) was often prescribed at discharge.

The objective of this study was to identify and quantify the reasons why ACS patients undergoing stenting at UNMH were prescribed lower-intensity dual antiplatelet therapy (DAPT) at discharge. Furthermore, the study aimed to identify practice patterns that could potentially lead to improved DAPT treatment for these patients.

## Methods

### Data Collection:

All studies were conducted with approval from the UNM Human Research Protections Office and Institutional Review Board. We reviewed electronic medical records and cardiac catheterization records of 326 patients who underwent PCI at UNMH between January 1, 2021, and June 30, 2022. These patients were identified from the American College of Cardiology (ACC) National Cardiovascular Data (NCDR) Cath PCI Registry, and the focus was on the subset of patients with ACS who survived until discharge (n = 229).

Demographic characteristics were collected for each patient, including gender and self-identified race. Clinical characteristics relevant to P2Y_12_ selection were obtained from a review of medical records such as bleeding risk, ischemic risk, co-morbidities, and concomitant medications. Pharmacists’ notes that documented their efforts to get appropriate insurance coverage and reasons for discharge on clopidogrel rather than ticagrelor were reviewed.

Patients discharged on aspirin and clopidogrel underwent review of medical records and cardiac catheterization lab records to determine if the discharge P2Y_12_ drug was appropriate. Criteria for appropriate therapy on clopidogrel were defined as: stable lesions in outpatients, stable lesions in patients hospitalized for a non-ischemic presentation, patients discharged on anti-coagulants in addition to P2Y_12_ inhibitors, patients with characteristics favoring clopidogrel over other P2Y_12_ inhibitors (e.g., high bleeding risk), patients who received thrombolytics before the procedure, and dyspnea before discharge attributed to ticagrelor.

Based on these data we categorized *procedural* P2Y_12_ administration as (1) appropriately given clopidogrel, (2) appropriately given ticagrelor, or (3) inappropriately given clopidogrel. We also categorized patients’ *discharge* P2Y_12_ as (1) appropriately on clopidogrel, (2) appropriately on ticagrelor, and (3) inappropriately on clopidogrel.

Patients that were discharged on clopidogrel inappropriately were further divided into categories according to rationale for not receiving appropriate therapy. Categories consisted of cost/insurance, patient preference, concern for daily adherence to a twice-daily medication (compared to a once-daily medication), and patients on clopidogrel before PCI and not switched to ticagrelor afterward.

### Statistical Analysis:

Observational data were reported as percentages and chi-square analyses were performed to compare demographic characteristics and co-morbidities. T-tests were performed to identify differences in continuous variables. P-values < 0.05 were considered statistically significant.

## Results

Out of our cohort patients undergoing PCI (n = 326) we identified 78 who underwent PCI for stable lesions (non-ACS) and 19 individuals who died during hospitalization. The remaining cohort (n = 229) were patients undergoing PCI for ACS who survived until discharge. Patients were characterized as average age 63.7 years, 24.9% female, 47.2% diabetic, and 88.6% medically insured at the time of the procedure. Self-identified ethnicity included 42.3% Caucasian non-Hispanic, 42.8% Hispanic, 7.0% American Indian/Native American, and 7.9% unspecified.

Regarding the administration of P2Y_12_ inhibitors at the time of the procedure for ACS patients, 17% (n = 39) received clopidogrel, 81.7% (n = 187) ticagrelor or ticagrelor with cangrelor, 0.9% (n = 2) received just cangrelor, and 0.4% (n = 1) received no P2Y_12_. Of the 39 patients that received clopidogrel at or before the time of the procedure 15.4% (n = 6) had an increased risk of bleeding, 30.8% (n = 12) were on or planned to be placed on anticoagulation, 2.6% (n = 1) were given clopidogrel at an outside hospital before arrival, 12.8% (n = 5) received thrombolytic therapy prior to arrival, and 38.5% (n = 15) were taking clopidogrel prior to the procedure. The patient that was not given a P2Y_12_ inhibitor was determined to be a candidate for coronary artery bypass by grafts during PCI by the operator and did not undergo stent placement. After PCI, 53.5% (100/187) of patients that were initially prescribed ticagrelor or ticagrelor with cangrelor at PCI were later switched to clopidogrel

We characterized patients at discharge as appropriately discharged on ticagrelor/prasugrel (38.0%, n = 87), appropriately discharged on clopidogrel (27.5%, n = 63), inappropriately discharged on clopidogrel (32.8%, n = 75), or not discharged on a P2Y_12_ inhibitor (1.7%, n = 4).

Of the patients that were appropriately discharged on clopidogrel, 26 were on an anticoagulant, 15 were given clopidogrel rather than more potent P2Y_12_ inhibitors due to their perceived increased risk of bleeding, 17 had dyspnea attributed to ticagrelor, and 5 were given thrombolytics prior to arrival for catheterization.

For patients inappropriately discharged on clopidogrel (n = 75), reasons included cost (n = 56), patient preference (n = 1), clinical inertia (taking clopidogrel before PCI and maintained on it afterward) (n = 17), and physician concern with adherence (n = 1).

Statistical analysis revealed no significant differences in sex, ethnicity, or age between patients appropriately discharged on ticagrelor and patients inappropriately discharged on clopidogrel or inappropriately discharged on clopidogrel solely due to cost.

Of the 162 patients that were deemed appropriate to receive ticagrelor/prasugrel following PCI, 71 identified as Hispanic, 95 identified as not Hispanic, and one individual declined to identify. We found no significant difference between patients self-identified as Hispanic that were treated with ticagrelor (45%) and patients that identified as non-Hispanic that were treated with ticagrelor (37%, p = 0.3).

Of the 162 patients that were deemed appropriate to receive ticagrelor/prasugrel following PCI, 141 had insurance and 21 did not. Patients with insurance were treated more often with ticagrelor when appropriate (60.3%) then patients without insurance (9.5%, P < .001).

## Discussion

The most important finding of this study is that of the cohort of 229 ACS patients who underwent stenting and survived until discharge, 24.5% were discharged on clopidogrel over ticagrelor due to cost despite ticagrelor offering greater protection from recurrent ischemic events. This highlights a significant portion of the population served by UNMH being unable to receive optimal medication. Notably, when ticagrelor was the appropriate discharge medication, patients with insurance were treated more often with it (60.2%) then patients without insurance (9.5%, P < .001). In 2021 11.6% of working-age adults in the United States did not have healthcare insurance[13]. This underscores the pressing need for improved healthcare insurance coverage for citizens of New Mexico and the necessity to address high prescription drug prices.

Our second most important finding is that 7.4% of ACS patients were discharged on clopidogrel rather than ticagrelor, with no reason identified other than prior usage. This suggests the presence of clinical inertia, where the healthcare team and/or patients opted to continue sub-optimal medication rather than switching to a more potent and effective P2Y_12_ inhibitor. While the exact motivation behind this clinical inertia cannot be determined from chart documentation, a simple intervention would be the education of physicians and patients about this phenomenon and encouraging the switch to a more appropriate P2Y_12_ inhibitor.

The third most important finding is that 7.4% of ACS patients were discharged on clopidogrel due to developing dyspnea attributed to ticagrelor during their inpatient hospital stay. Most episodes of ticagrelor-induced dyspnea are transient and last for less than a week.[14] Healthcare professionals can engage in discussions with patients and make efforts to determine if the patient can tolerate and overcome the temporary dyspnea associated with ticagrelor.

Ticagrelor is currently accepted as a superior P2Y_12_ inhibitor treatment in patients who have undergone PCI for ACS, based largely on a randomized controlled trial[2] and non-randomized cohort studies[4–6]. This finding has been reinforced by a 2020 meta-analysis of 52,816 patients that found a decreased risk of mortality and ischemic events in ticagrelor over clopidogrel[15]. Other studies have produced conflicting results. A retrospective cohort study of 31,290 individuals showed no benefit to taking ticagrelor but noted an increased risk of hemorrhagic events and dyspnea[16]. A systematic review and meta-analysis of 18,365 patients in 2021 found similar results[17]. Although these studies have methodologic flaws, advances after the original ticagrelor randomized controlled trials such as second-generation drug-eluting stents, could account for ticagrelor not outperforming clopidogrel in more recent studies[16]. Regardless, until revision, current guidelines for the care of patients recommend ticagrelor for patients that have undergone PCI for ACS.

### Limitations:

Pharmacists’ notes at the time of discharge usually documented their efforts to get appropriate insurance coverage and reasons for discharge on clopidogrel rather than the ticagrelor recommended by discharging cardiologists. However, this documentation was ad hoc, not formalized.

Data on income, education, employment status, and family stability were not available in the medical record or the American College of Cardiology National Cardiovascular CathPCI Registry. This prevented determining if other socioeconomic determinants of health (other than insurance status) affected discharge P2Y_12_ therapy.

## Conclusions

UNMH is critical safety-net hospital for the state of New Mexico. This study has identified ways in which patients receiving care at UNMH for ACS could receive sub-optimal DAPT care after PCI so that they may be optimized. Increasing financially restricted access to ticagrelor could help up to 24.5% of patients reduce the risk of ischemic events. Ensuring proper updates to pre-existing medications at the time of discharge could potentially improve the outcome in 7.4% of patients. 7.4% of patients could potentially have their outcomes improved by taking the time to talk them through their transient dyspnea. Our findings strongly suggest that expanding healthcare insurance coverage to individuals would assist in achieving the goal of decreasing sub-optimal DAPT coverage.

## Figures and Tables

**Table 1:** Discharge P2Y_12_ Therapy for 229 Patients who underwent PCI for ACS at The University of New Mexico Hospital from 1/1/21 through 6/30/22 and survived until discharge (n=229)

Rational for Discharge P2Y_12_	Method of Identification
**Appropriate Discharge on Clopidogrel (n=63)**	
Requirement for anti-coagulation (n =26)	Discharge on anti-coagulant (confirmed in medical record)
Risk/benefit favored clopidogrel (n =15)	High bleeding risk (specifically mentioned in the medical record)
Dyspnea before discharge attributed to ticagrelor (n =17)	Dyspnea before discharge was attributed to ticagrelor (specifically mentioned in the medical record)
Given thrombolytics prior to coronary intervention (n=5)	Thrombolytics for ST elevation myocardial infarction prior to arrival at University of New Mexico Hospital
**Sub-Optimal Discharge on Clopidogrel (n=75)**	
Cost (n =56)	Acute coronary syndrome but cost identified by inpatient service pharmacologist as barrier
Patient preference (n =1)	Specified in the medical record
Admitted on clopidogrel (n =17)	Medical records document chronic clopidogrel prior to admission, no other explanation found after extensive review of medical record
Concern for adherence (1)	Patient prescribed clopidogrel over ticagrelor due to concern for adherence to twice daily medication
**Appropriate Discharge on Ticagrelor/Prasugrel (n =87)**	
**Not discharged on a P2Y_12_ inhibitor (n=4)**	

**Table 2.** Characteristics of Patients Discharged on Clopidogrel Inappropriately vs Ticagrelor Appropriately

	Column 1	Column 2	Column 3	Column 4	Column 5
	Ticagrelor Appropriately	Clopidogrel Inappropriately	P (Col 1 vs Col 2)	Inappropriate-cost/insurance	P (Col 1 vs 4)
Female	22% (19/87)	21% 16/75	0.94	16% (9/56)	0.40
Age, (SD)	63.1 (11.09)	62.6 (11.03)	0.76	62.85 (SD = 11.41)	0.95
With DM	52.9% (46/87)	42.7% (32/75)	0.062	37.5% (21/56)	0.023
With Insurance	97.7% (85/87)	74.7% (56/75)	1.14 X10^−5	71.4% (40/56)	3.7 X10^−6
Caucasian non-Hispanic	37.9% (33/87)	48% (36/75)	0.26	50% (28/56)	0.15
Hispanic	44.8% (39/87)	37.3% (28/75)	0.334	47.8% (22/56)	0.51
American Indian	8.0% (7/87)	8% (6/75)	0.99	5.3% (3/56)	0.54
